# EGR1 dysregulation defines an inflammatory and leukemic program in cell trajectory of human-aged hematopoietic stem cells (HSC)

**DOI:** 10.1186/s13287-021-02498-0

**Published:** 2021-07-22

**Authors:** Christophe Desterke, Annelise Bennaceur-Griscelli, Ali G. Turhan

**Affiliations:** 1 INSERM UA9, University Paris-Saclay, 94800 Villejuif, France; 2 ESTeam Paris Sud, INGESTEM National IPSC Infrastructure, University Paris-Saclay, 94800 Villejuif, France; 3 Division of Hematology, APHP-Paris Saclay University Hospitals, Le Kremlin Bicêtre 94275, 94800 Villejuif, France; 4 Faculty of Medicine, University Paris Saclay, 94275 Le Kremlin Bicêtre, France

**Keywords:** Hematopoietic stem cell, EGR1, Aging, Inflammation, Leukemia, Single cell

## Abstract

**Background:**

During aging, hematopoietic stem cells (HSC) lose progressively both their self-renewal and differentiation potential. The precise molecular mechanisms of this phenomenon are not well established. To uncover the molecular events underlying this event, we have performed a bioinformatics analysis of 650 single-cell transcriptomes.

**Methods:**

Single-cell transcriptome analyses of expression heterogeneity, cell cycle, and cell trajectory in human cell compartment enriched in hematopoietic stem cell compartment were investigated in the bone marrow according to the age of the donors. Identification of aging-related nodules was identified by weighted correlation network analysis in this primitive compartment.

**Results:**

The analysis of single-cell transcriptomes allowed to uncover a major upregulation of EGR1 in human-aged lineage−CD34+CD38− cells which present cell cycle dysregulation with reduction of G2/M phase according to less expression of CCND2 during S phase. EGR1 upregulation in aging hematopoietic stem cells was found to be independent of cell cycle phases and gender. EGR1 expression trajectory in aged HSC highlighted a signature enriched in hematopoietic and immune disorders with the best induction of AP-1 complex and quiescence regulators such as EGR1, BTG2, JUNB, and NR41A. Sonic Hedgehog-related TMEM107 transmembrane molecule followed also EGR1 cell trajectory. EGR1-dependent gene weighted network analysis in human HSC-associated IER2 target protein-specific regulators of PP2A activity, IL1B, TNFSF10 ligands, and CD69, SELP membrane molecules in old HSC module with immune and leukemogenic signature. In contrast, for young HSC which were found with different cell cycle phase progression, its specific module highlighted upregulation of HIF1A hypoxic factor, PDE4B immune marker, DRAK2 (STK17B) T cell apoptosis regulator, and MYADM myeloid-associated marker.

**Conclusion:**

EGR1 was found to be connected to the aging of human HSC and highlighted a specific cell trajectory contributing to the dysregulation of an inflammatory and leukemia-related transcriptional program in aged human HSCs. EGR1 and its program were found to be connected to the aging of human HSC with dissociation of quiescence property and cell cycle phase progression in this primitive hematopoietic compartment.

**Supplementary Information:**

The online version contains supplementary material available at 10.1186/s13287-021-02498-0.

## Background

Aged blood cells display different functional aberrations depending on their cell type, which might lead to the development of hematological disorders such as leukemia, anemia, and declining immunity [1]. At the adult stage, the hematopoietic organ is mainly composed of hematopoietic cells and their niche is principally localized in the bone marrow. Some cytokines such as  interleukin 1-beta in the bone marrow microenvironment are known to contribute to the aging process in the hematopoietic stem cell (HSC) compartment [2]. The aged bone marrow also has a reduced capability of retaining HSCs in their niche with increased hematopoietic stem cell mobilization [3]. The main discovery concerning hematopoietic stem cell aging highlighted that HSCs from old mice had only 25% of the efficiency of young HSCs in terms of homing and engraftment into the bone marrow of a transplant recipient [4]. Hematopoietic stem cells exhibit age-related changes that include impaired adherence to stromal cells, and in some strains of mice [5] as well as  in elderly humans, quantitative and qualitative changes with a  myeloid bias in terms of their differentiation potential [6]. Hematopoietic aging is also accompanied by reduced adaptive immune responses [7], increased susceptibility to develop anemia [8], and susceptibility to developed myeloid clonal diseases [9]. Most hematopoietic stem cells are quiescent and rarely enter the cell cycle to self-renew or differentiate to maintain hematopoiesis under homeostasis [10]. The number of hematopoietic stem cells is found to be increased in the bone marrow with the age in some strains of mice [11].

Previous work aiming to characterize the aged hematopoietic stem cell transcriptome analyses has used bulk and/or single-cell (sc)RNA-sequencing to compare cell surface marker-defined young and old HSCs,  has identified signatures indicating increased HSC self-renewal and altered cell cycle [12–17]. At the cell cycle level, in murine models, it has been shown that long-term hematopoietic stem cells were significantly underrepresented among cells in the G1/S cell cycle phase [14]. Behind this aging-related phenotype molecular changes in the genome, transcriptome and epigenome have been characterized. With DNA analyses, some clonal alteration has been observed during aging process of hematopoiesis. In young healthy donors, all HSCs are equal in their capabilities to produce all the mature blood cells and their pool maintains polyclonal hematopoiesis [18]. At the epigenetic level, the chromatin structure of hematopoietic stem cells is revealed by a single-cell ATAC-sequencing, showing its role in the execution of the different modes of HSC division. Old HSCs were shown to divide symmetrically and supporting self-renewal, whereas young HSCs preferentially underwent asymmetric division,  generating progenitor cells [19]. Multimodal epigenetics analyses at a single-cell level pinpointed that chromatin modification profiles in younger individuals are more homogenous than those of older ones [20]. Understanding hematopoietic stem cell heterogeneity and its modifications during aging at the single-cell level could help understanding the molecular mechanism implicated. This understanding could help to correct the bias of hematopoiesis observed during aging and to develop strategies to reduce the occurrence of a hematopoietic disorder.

We report in this work a comprehensive analysis of single-cell transcriptome (*n* = 650) correlated with age in hematopoietic cells. We found that EGR1 upregulation was connected to the aging of human HSC and highlighted a specific cell trajectory contributing to the dysregulation of an inflammatory and leukemia-related transcriptional program in aged human HSCs. EGR1 deregulation in this cell trajectory was found independent of cell cycle phase progression suggesting a dissociation of quiescence property and cell cycle phase progression in the human primitive hematopoietic compartment. This EGR1-regulated program was confirmed to be highly connected to age during gene network analysis of CD34+CD38− lineage− in human HSCs.

## Methods

### Public datasets

#### Single-cell transcriptome of human hematopoietic stem cell-enriched compartment (GSE104379)

In order to investigate expression heterogeneity in human hematopoietic stem cell compartment according to the age of donors, the single-cell Transcriptome Dataset GSE104379 performed on human hematopoietic stem cell-enriched cell compartment was downloaded to be processed single-cell object in R software environment. This dataset comprised 650 single-cell transcriptomes.

After single-cell separation using Fluidigm C1 system, cDNA was made with Clontech SMARTer® Kit and Libraries were constructed using Nextera XT DNA Library Preparation Kit. Reads were sequenced on Illumina HiSeq 2500 and aligned on HG19 human genome. Transcript by million (TPM) was quantified and incremented before log2 transformation [21].

#### Transcriptome RNA sequencing of human hematopoietic stem cell (GSE104406)

RNA-sequencing was also performed on human HSC (Lineage−, CD34+, CD38−) purified from 10 young and 10 old healthy donors. RNAseq was performed on FACS-isolated human bone marrow-derived HSC in young (18–30years old) and old  (65–75) donors. Starting from RNA samples, ribosomal RNA depletion was performed before the synthesis of stranded libraries using the SMARTer Stranded RNA-seq Kit. Libraries were sequenced on the HiSeq-2500 with 50 bp pair-end sequencing. After trimming, reads were aligned on the HG19 human genome and counts were normalized by rlog transformation batch correction [21].

#### Microarray datasets for meta-analysis on human long-term HSC compartment

In order to confirm the deregulation of EGR1, a selection of transcriptome datasets performed on different hematopoietic compartments in human hematopoiesis was investigated through the Geo Expression Omnibus web portal. This selection comprised datasets (GSE35010, GSE35008) of human bone marrow long-term HSC from healthy donors and acute myeloid leukemia (AML) patients [22, 23], but also a dataset including  HSC with long-term repopulation potential from the human cord blood in xenotransplantation assays (GSE58299) [24]. In addition, the selection included a dataset comprising long-term HSC compartment from different tissues (bone marrow and cord blood) from healthy donors and cells of different hematopoietic compartments from AML patients (GSE24006) [25]. Transcriptome meta-analysis was performed after batch correction with Combat algorithm [26].

#### Bioinformatics analyses

Bioinformatics analyses were performed with R software environment version 4.0.2 and Rstudio version 1.1.383 software. Graphs were performed with ggplot2 graph definition with R-package version 3.3.2 [27]. Functional enrichments on gene lists were performed with Toppgene online application by inference on different databases such as Gene Ontology and DisGeNet [28].

#### Single-cell transcriptome analyses

Single-cell transcriptome matrix and cell phenotypes were integrated to create a Seurat object with Seurat R-package version 3.2.2 [29]. Cell pre-processing was performed with the threshold of filtration applied to cells expressing a minimum of 200 features. Most variable features were detected by applying variance stabilization transformation (vst) algorithm, and data were scaled according to these variable features. Unsupervised dimension reduction by principal component analysis (PCA) according to variable features and applied on 50 components. Subsequently, t-SNE dimension reduction was performed by taking into account the 20 first components of PCA after ElbowPlot assessment. Feature visualization was performed with different Seurat graphs like Dimplot, Ridgeplot, ViolinPlot, and FeaturePlot. For cell cycle analysis in Seurat object, PCA was run on the data with geneset specific of S and G2M cell cycle phases defined upon aging on hematopoietic stem cell by Tirosh et al. [14]. To reconstitute, EGR1 cell trajectory matrix and cell phenotype were integrated with a monocle single-cell object in monocle R-Bioconductor package version 2.16.0 [30]. Cell hierarchy was defined on EGR1 expression level in 3 cell groups: cells with EGR1 low expression under threshold 3, cells with medium expression of EGR1 between 3 and 8 and cells with high expression over 8. Unsupervised dimension reduction by t-SNE analysis was run on 30 first components. After cell filtration based on a minimum number of gene expression, a differential expression analysis was run based on defined cell hierarchy. Cells were ordered according to this test to performed pseudotime transformation by running DDRtree algorithm. The trajectory was reconstitute visually with plot_cell_trajectory() monocle function and pseudotime heatmap was drawn with 75 significant genes found on EGR1 trajectory. Gene pseudotime regulation graph was built with plot_genes_in_pseudotime() function.

#### RNA-sequencing analyses

Normalized matrix by rlog transformation and with batch correction on dataset GSE104406 comprising HSC analysis of 10 young donors and 10 old donors was used to performed supervised analysis in limma R-bioconductor package version 3.44.3 [31]. Supervised differential expression analysis between young and old HSCs was performed with False Discovery Benjamini–Hochberg adjustment and Bayesian correction and supervised Volcanoplot was drawn. In order to understand predictive score of differential expressed genes, a supervised learning by leave-one-out procedure was performed with Pamr package version 1.56.1 [32] based on Shrunken Centroid identification.

#### Network analyses

It was performed according to the algorithm developed by Horvath and co-workers [33] with WGCNA R-package version 1.69 and using HSCs EGR1 trajectory geneset intercept on GSE104406 dataset analysis. Assessment of the geneset input in WGCNA analysis was driven with the characterization of the power ß obtained from the scale-free fit model curve (r2 > 0.9); 44 as the first power value reaching R^2^ > 0.9. The simplest module definition was defined by clustering using the Topological Overlap Matrix (TOM) and verified by multidimensional scaling by checking relevant module sizes for identified ones. For the Cytoscape (software version 3.6.0), connectivity plot [34] edges were selected above a given weight (intra-modular topological overlap measure) threshold.

## Results

### Old human HSCs present deregulation of CDK4/6/D-type cyclin CCND2 complex and a high expression of EGR1

EGR1 transcription factor is known to be upregulated in cyclin-dependent kinase 6 (CDK6) deficient HSC which do not efficiently repopulate upon competitive transplantation assay and more susceptible to 5-fluorouracil (5FU) treatment due to their committed nature. Activation of HSCs requires CDK6, a cell cycle regulator which interferes with some key transcription factors, including EGR1 [35].

**Fig. 1:**
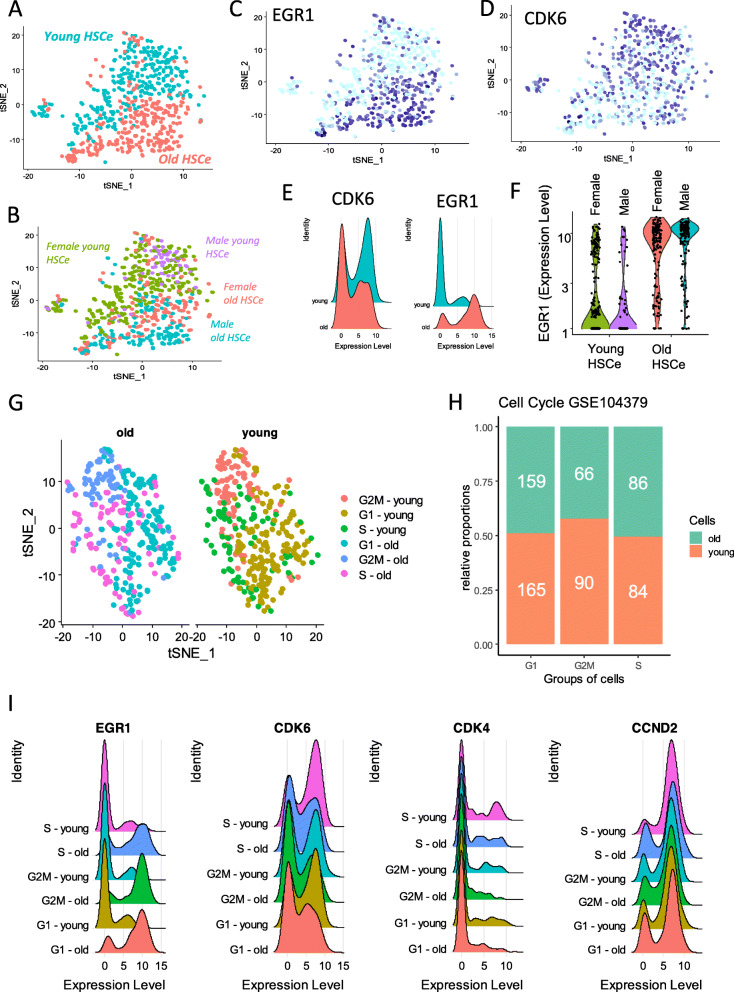
EGR1 transcription factor is overexpressed in aged human hematopoietic stem cell-enriched compartment: from **A** to **I**: single-cell analysis performed on dataset GSE104379 processing HSC-enriched compartment (HSC) equivalent to lineage –/CD34+/CD38– human cell compartment; **A** tSNE analysis of young and old HSC single-cell transcriptome, **B** tSNE analysis of young and old HSC single-cell transcriptome stratified by genders; **C** expression level of EGR1 in tSNE analysis of HSC; **D** expression level of CDK6 in tSNE analysis of HSC; **E** ridge plot comparing expression of CDK6 and EGR1 stratified on the age of donors; **F** violin plot of EGR1 expression stratified on age and gender of donors; **G** cell cycle analysis stratified on age of donors; **H** barplot of cell cycle phase stratified according to the age of donors (white numbers represent the number of cells classified in each phase); **I** ridgeplot of CDK4, CDK6, and CCND2 expressions according to cell cycle phases according age of donors

The single-cell transcriptome of the human hematopoietic stem cell-enriched compartment (HSC) from the bone marrow was investigated through the GSE104379 dataset. This dataset comprised transcriptomes of 650 bone marrow HSC coming from 11 healthy donors (young *n* = 5, (age range: 24–37) and old *n* = 6 donors (age range: 64–71). After pre-processing single-cell data, normalization and scaling of the data steps were performed in order to process dimensional reduction with PCA and tSNE algorithms. Based on the total transcriptome, the first projection map of tSNE reduction showed a good discrimination between HSC from old donors and HSC from young donors (Fig. 1a). Gender stratification of this analysis did not show an effect of this parameter (Fig. 1b). With regard to age analysis (Fig. 1a), EGR1 expression (Fig. 1c) seemed to be higher in HSC from older donors. In contrast, CDK6 expression exhibited the inverse pattern of expression with higher levels in HSC from younger donors (Fig. 1d). This is confirmed by comparative ridgeplot analysis showing that expression of EGR1 is mainly in older cells and expression of CDK6 mainly in younger cells (Fig. 1e). High expression of EGR1 in older HSC was not found to be dependent of the gender factor (Fig. 1e). CDK6 belongs to an important complex with cyclins D which promotes the G1 to S phase progression through the cell cycle [36]. Single-cell transcriptome analysis of cell-cycle scoring was investigated in HSC according to the age of donor parameter [14]. After unsupervised principal component analysis restricted to the cell cycle markers, tSNE analysis allowed stratified cell phases according to the age of donors (Fig. 1g). This analysis revealed a reduction of G2M phase proportion in old HSC (Fig. 1h) suggesting dysregulation of cell cycle mechanisms in old HSC as compared to the young ones. Cell cycle analysis also revealed that EGR1, CDK6, and CDK4 regulations of transcripts were found independent of phase characterization (Fig. 1i). In contrast, among D-type cyclins, CCND2, the best expressed one in HSC, was found less positive in S phase of old HSC. These results suggest that old human HSC are characterized by deregulation of CDK4/6/D-type cyclin CCND2 complex and harbored an increased expression of an EGR1 transcription factor.

### EGR1 expression heterogeneity with age defines a specific cell trajectory in human HSC

**Fig. 2:**
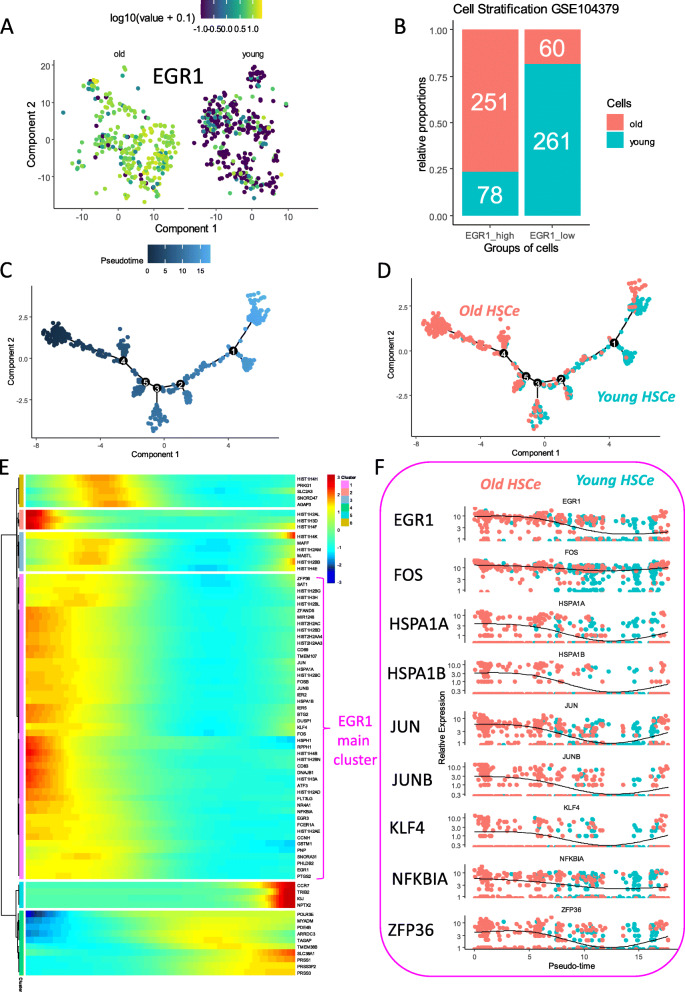
During aging, EGR1 cell trajectory in human HSC shows an activation of transcription factors implicated in inflammation: from **A** to **F**: single-cell analysis performed on dataset GSE104379 processing HSC-enriched compartment (HSC) equivalent to lineage –/CD34+/CD38– human cell compartment: **A** EGR1 expression in t-SNE analysis stratified on age groups, **B** barplot of cell stratification between age group and EGR1 expression relative groups (EGR1 low and EGR1 high expression with threshold on value of 3), **C** single-cell pseudotime transformation based on EGR1 expression, **D** EGR1 expression-based cell trajectory stratified on groups of age, **E** pseudotime heatmap of best markers which followed pseudotime transformation based on EGR1 expression level in HSC, and **F** some transcription factors and molecules found in EGR1 cluster after pseudotime transformation

Connected to cycle scoring deregulation in HSC according to age, EGR1 was found to be overexpressed in HSC from old donors as compared to ones from young donors. EGR1-CDK6 axis is important for the control of exit from quiescence of HSCs [35]. In this work, EGR1 upregulation in old HSC (Fig. 2a) was observed so an EGR1-dependent cell trajectory was built according to the age parameter of the donors. Based on EGR1 expression threshold definition at level of 3 (Fig. 1f), HSC single-cell transcriptome of dataset GSE104379 was stratified in two cell groups: a group of cells with high expression of EGR1 (quantification over 3) comprising a set of 329 cells and a group of cells with low expression of EGR1 (quantification under 3) comprising a set of 321 cells. The analysis according to age in these groups confirmed an important proportion of aged HSC in the EGR1_High group and as compared to the important proportion of young HSC in EGR1_low group (Pearson’s chi-squared test, *p* value < 2.2e−16, Fig. 2b). According to the EGR1 cell groups, differential gene expression analysis was performed (supplemental table 1) using pseudotime transformation (Fig. 2c) with DDRtree dimensional reduction algorithm in monocle2 R-package.

This pseudotime transformation revealed a good stratification of age-related cell groups on EGR1-dependent cell trajectory (Fig. 2d) suggesting the important influence of EGR1 in aged human HSC. Clustering analysis on signature of EGR1 cell trajectory confirmed a main cluster of molecules following expression heterogeneity of EGR1 human HSC (Fig. 2e, pink cluster). Among this EGR1 trajectory cluster, we have identified quiescence regulators such as JUNB, BTG2, NR4A1, and AP1-complex molecules such as JUNB and DUSP1. Some membrane molecules such as TMEM107 and CD69 as well as a chaperone molecule HSPA1B were upregulated. Similarly, an inflammation mediator NFKBIA and one miRNA MIR1248 were confirmed to be regulated in old HSC as compared to young HSC (supplemental figure 1).

Functional enrichment performed on Gene Ontology Biological Process showed implication of some of these molecules in hematopoietic or lymphoid organ development (false discovery rate *q* value = 1.312E−6, Fig. 2f). Functional enrichment performed on DisGeNet disease database with molecules belonging to the EGR1 trajectory cluster highlighted a network of molecules implicated in the pathophysiology of hematopoietic disorders (lymphoid and myeloid disorders, supplemental figure 2A). The central role of JUN, GSTM1, NR4A1, FOSB, FOS, EGR1, ZFP36, and KLF4 in pathophysiological in these hematopoietic disorders was highlighted by network analysis (supplemental figure 2B). This disease-associated functional enrichment also revealed that EGR1 trajectory cluster component could be also implicated immune disorders principally such as autoimmune diseases, asthma, colitis, and arthritis (supplemental figure 3A). Network analysis on immune disorder enrichment placed PTGS2, DUSP1, NR4A1, FOSB, FOS, PNP, JUN, ZFP36, and KLF4 with a central role in these diseases.

### EGR1-dependent weighted gene network analysis identify specific gene modules associated with the aged phenotype

**Fig. 3:**
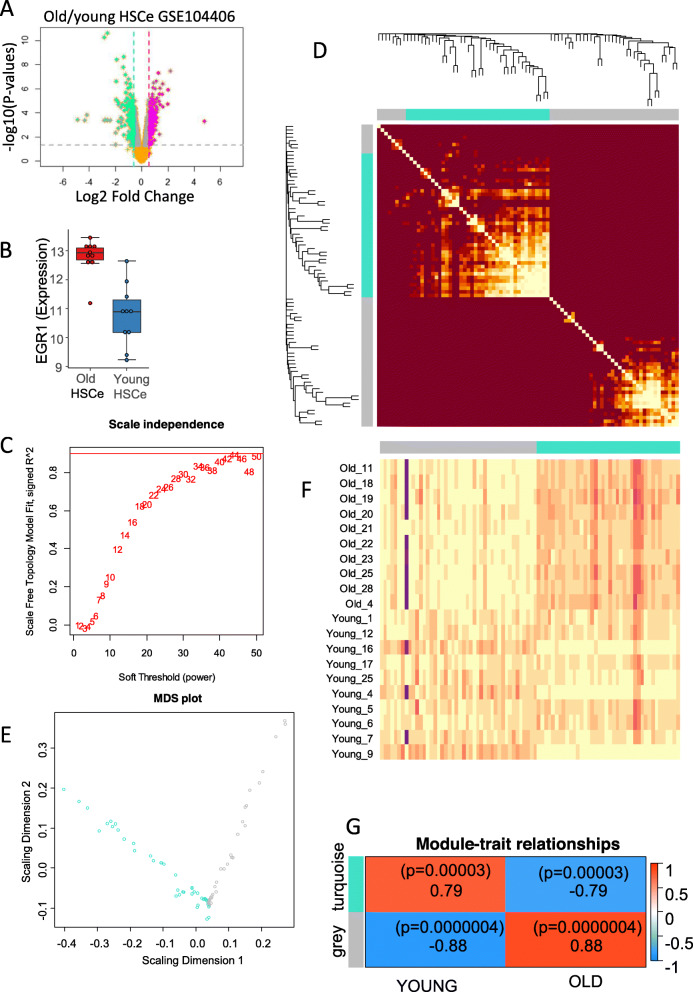
EGR1 gene connected network confirms the phenotype relation with age in HSC human cell compartment: from **A** to **E** process of RNA-sequencing from dataset GSE104406; **A** volcano plot of differential expressed genes in RNA-sequencing transcriptome of old HSC versus young HSC, **B** expression level of EGR1 stratified on age groups, **C** power analysis for WGCNA analysis performed on EGR1-dependent signature in human HSC, **D** TOM topological heatmap of gene modules identified during WGCNA analysis with EGR1-dependent signature in human HSC, **E** multidimensional scaling plot of genes classed by modules during WGCNA analysis, **F** expression heatmap of genes stratified by modules during WGCNA analysis, and **G** module correlation to phenotype (correlation score with *p* as relative *p* values)

**Fig. 4:**
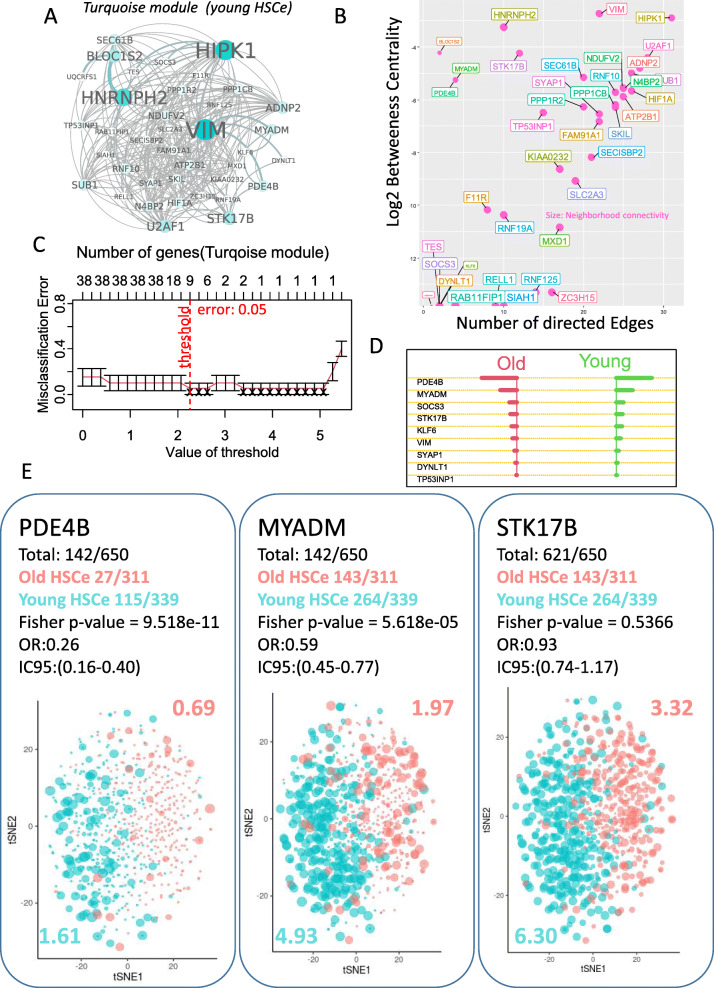
EGR1-dependent young-specific gene network module in human HSC: **A** Gene connections identified in young HSC compartment during EGR1-dependent WGCNA analysis (node and color nodes are proportional of node centrality, color, and size of the edge are proportional of edge centrality); **B** Network analysis in young HSC module with a number of direct edges on the first axis and logarithm base 10 of betweenness centrality and neighborhood connectivity for the size of the gene points; **C** misclassification error graph obtained in age supervised machine learning with genes of young HSC module; **D** barplot of predictive scores for predictive markers of young HSC; **E** single-cell expression of PDE4B, MYADM, and STK17B in human HSC stratified on age of donors (Fisher’s exact test was employed between the amount of positive cells in each group, numbers of positive cells, and mean expression of each marker are written in the corresponding color of groups)

**Fig. 5:**
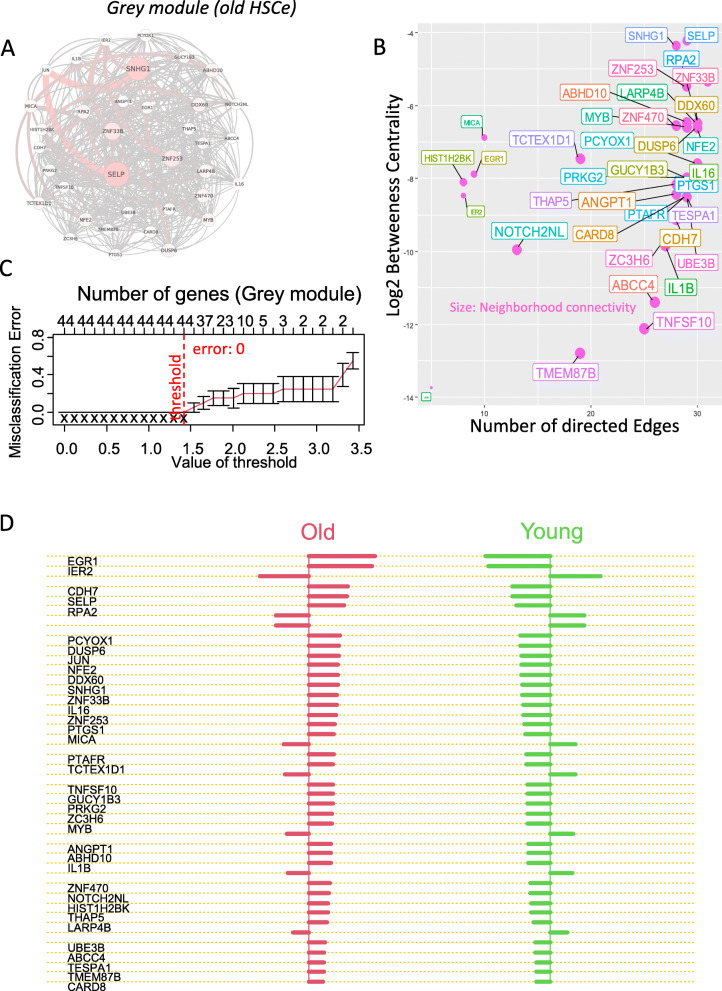
EGR1-dependent old-specific gene network module in human HSC: **A** Gene connections identified in old HSC compartment during EGR1-dependent WGCNA analysis (node and color nodes are proportional of node centrality, color, and size of the edge are proportional of edge centrality); **B** Network analysis in old HSC module with a number of direct edges on the first axis and logarithm base 10 of betweeness centrality and neighborhood connectivity for the size of the gene points; **C** misclassification error graph obtained in age-supervised machine learning with genes of old HSC module; **D** barplot of predictive scores for predictive markers of young HSC; and **E** expression dotplot showing in individual samples the expression of best predictive markers of old HSC

**Fig. 6:**
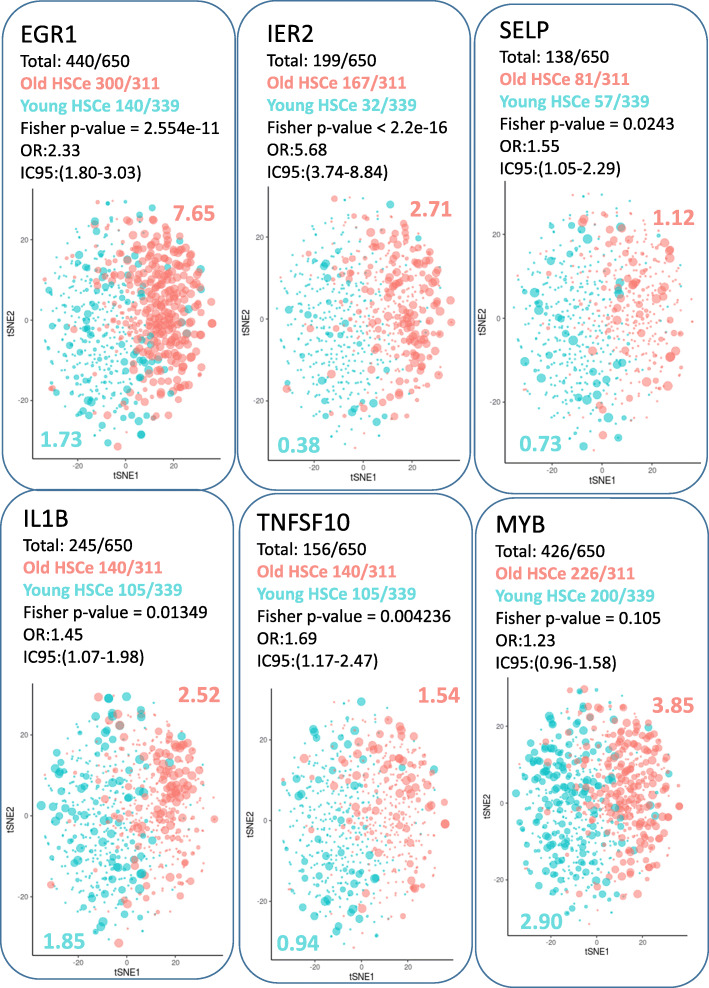
Single-cell expression analyses of markers belonging to old HSC connected module: Single-cell expression of EGR1, IER2, SELP, IL1B, TNFS10, and MYB in human HSC stratified on the age of donors (Fisher’s exact test was employed between the amount of positive cells in each group, numbers of positive cells and mean expression of each marker are written in the corresponding color of groups)

**Fig. 7:**
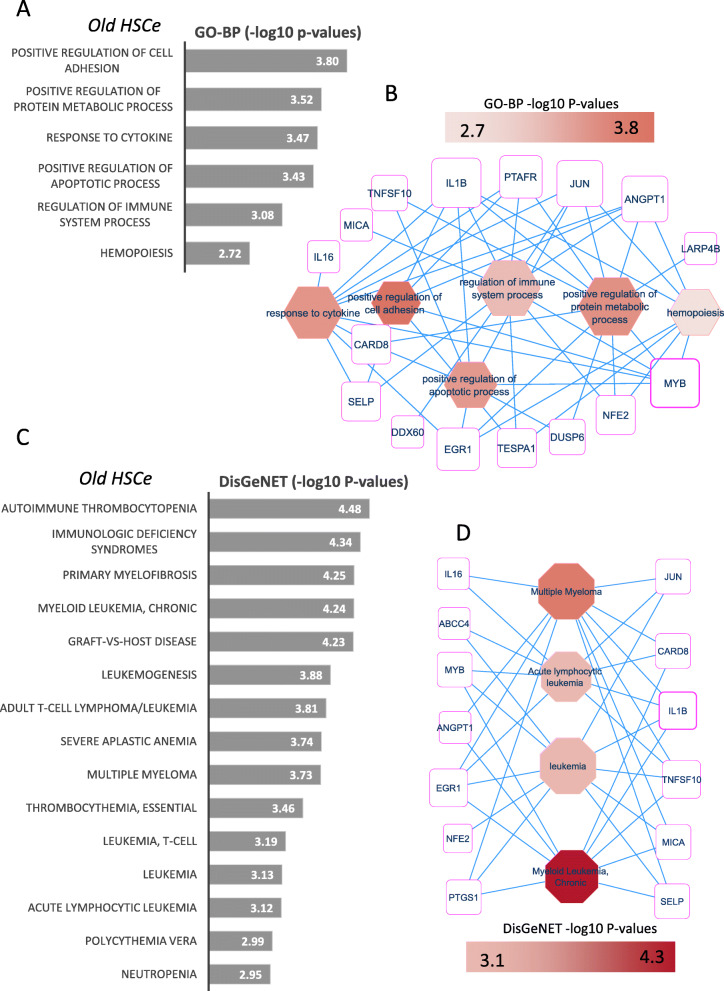
Leukemogenesis signature enriched in gene module of old human HSC: **A** barplot of functional enrichment performed on Gene Ontology Biological Process database (scale in negative logarithm base 10 of the *p* values), **B** functional enrichment network performed with genes from old HSC module which were enriched in main biological processes, **C** barplot of functional enrichment performed on DisGeNET database (scale in negative logarithm base 10 of the *p* values), and **D** functional enrichment network performed with genes from old HSC module which were enriched in main hematopoietic disorders

In order to find gene-regulated modules at the cell system level, a WGCNA analysis [33] was performed with the EGR1 trajectory signature on the human HSC RNA-sequencing dataset (GSE104406). Differential expression gene (DEG) analysis between young and old donor samples was performed on rlog counts (Fig. 3a, Supplemental table 2). In this independent dataset performed on human HSC, upregulation of EGR1 was confirmed in old HSC as compared to young ones (adjusted *p* value = 8.28e−4, Fig. 3b). These DEG were intercepted with a single-cell EGR1 cell trajectory analysis (Supplemental table 1). WGCNA analysis revealed a topological saturation to a power value of 44 (Fig. 3c) which allowed to class two main gene clusters (turquoise and gray) on heatmap of Topology Overlap Matrix (Fig. 3d) and contributive genes of in both modules are well stratified by multidimensional scaling (Fig. 3e). Expression heatmap of selected gene from modules harbored different expression profiles according to HSC phenotype (Fig. 3f). A significant correlation was found between a turquoise module and young HSC phenotype (*r* = 079, *p* value = 3e−5, Fig. 3g) and between gray module and old HSC phenotype (*r* = 0.88, *p* value = 4e−7, Fig. 3g). These results suggest that the EGR1 expression trajectory could be associated with the regulation of specific gene modules according to the age of the cells in the human HSC cell compartment. Turquoise gene module (Figs. 3g and 4a) associated with young HSC phenotype revealed a high-connected network around 38 genes (Fig. 4a, b). Supervised machine learning attributed best young HSC predictive scores (Fig. 4c, d, supplemental table 3) for PDE4B, MYADM, and STK17B in RNAseq dataset GSE104406 (supplemental figure 4A) and verified to be regulated between young and old HSC at the single-cell level in dataset GSE104379 (Fig. 4e). Also in a turquoise module, single-cell regulation of HIF1A and KLF6 was confirmed to have higher expression in young HSC as compared to old HSC (supplemental figure 4B). Six predictive markers of young HSC phenotype with weaker expression were confirmed to be regulated at the single-cell level: TP53INP1, KIAA0232, HNRNPH2, SECISBP2 DYNLT1, and ATP2B1 (supplemental figure 5). Gray gene module associated with old HSC phenotype revealed a high connected module around 36 genes (Fig 5a, b). Supervised machine learning allowed attribution of  best old HSC predictive scores (Fig. 5c, d, supplemental table 4). Among predictive molecules from gray module in RNAseq dataset GSE104406, like EGR1, IER2, SELP, IL1B, TNFSF10, and MYB (Fig. 6) and DUSP6, NFE2, ANGPT1, PTGS1, CARD8, and ZC3H6 (supplemental figure 6) were confirmed to be upregulated between young and old HSC at single-cell level in dataset GSE104379. Gene functional enrichment performed with gray module genes associated to old HSC phenotype allowed to highlight the implication of some of these molecules in important biological processes such as  regulation of cell adhesion, protein metabolism, response to cytokines, regulation of the immune system, and hemopoiesis (Fig. 7a, b). Also, enrichment on Disease database DisGeNet with gray module genes confirmed the implication of some of these molecules (JUN, IL16, ABCC4, CARD8, MYB, IL1B, ANGPT1, EGR1, TNFSF10, NFE2, MICA, PTGS1, and SELP) in the pathophysiology of leukemia (Fig. 7c, d). These results suggest that gene module discovery in the old HSC phenotype connected to a higher expression of EGR1 contained molecules implicated in cell homeostasis and leukemia pathophysiology.

### EGR1 is upregulated with age in short- and long-term human hematopoietic stem cells

**Fig. 8:**
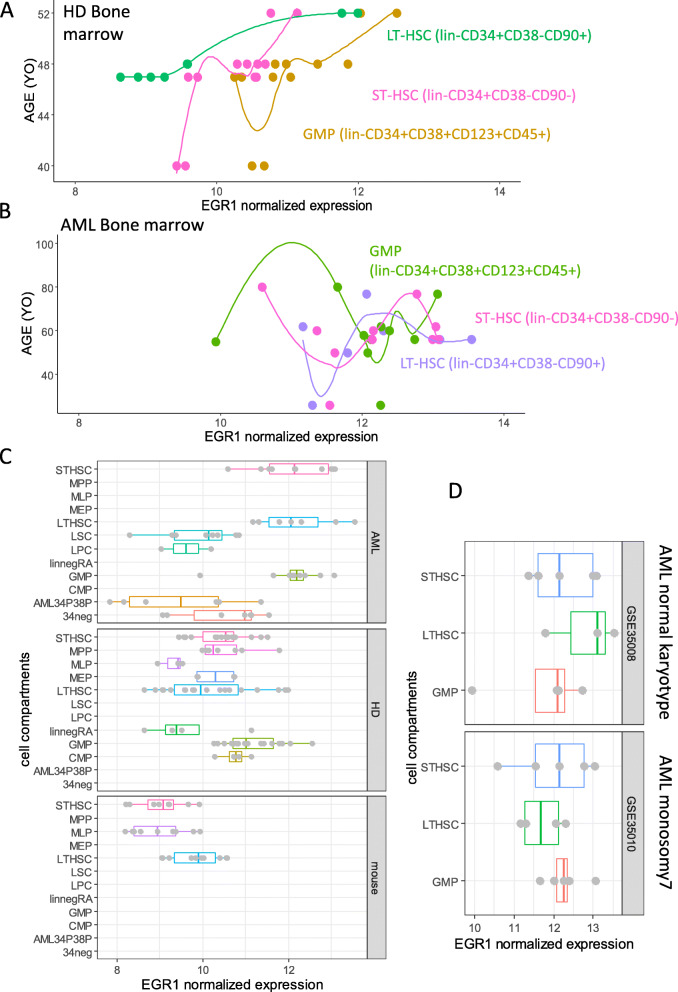
EGR1 expression and age relations in human long-term hematopoietic stem cells of healthy donors and that of acute myeloid leukemia patients: **A** scatterplot of age versus EGR1 expression in human bone marrow hematopoietic compartments of healthy donors; **B** scatterplot of age versus EGR1 expression in human bone marrow hematopoietic compartments of healthy donors; **C** boxplot of EGR1 expression in the different hematopoietic compartment (STHSC, short-term HSC; MPP, multipotent progenitor; MLP, multi-lymphoid progenitor; MEP, megakaryocyte erythrocyte progenitor; LTHSC, long-term HSC; LSC, leukemic stem cell; LPC, leukemic progenitor cell; linnegRA, Lin-CD34+CD38-CD90-CD45RA+ from cord blood; GMP, granulocyte myeloid progenitor; CMP, common myeloid progenitor; AML34P38P, compartment CD34+CD38+ of AML samples; 34neg, compartment of CD34− cells; **D** Boxplot of EGR1 expression in LT-HSC, ST-HSC, and GMP stratified on karyotype status (normal versus monosomy 7)

In order to connect  deregulation of EGR1 with age of human HSCs, a transcriptome meta-analysis was investigated in the human primitive hematopoietic compartment which has been characterized by functional assays. Distinct human dataset of the transcriptome was merged and normalized on their batch heterogeneities (Supplemental Figure 7). After the elimination of experimental bias, we could observe in healthy bone marrow samples a progressive increase of EGR1 expression with the age of the donors in the distinct hematopoietic compartments: long-term HSC (LT-HSC), short-term HSC (ST-HSC), and granulocyte myeloid progenitors (GMP) (Fig. 8a).

In AML bone marrow samples (Fig. 8b), expression of EGR1 was found to be higher in LT-HSC, ST-HSC, and GMP as compared to their respective similar compartment in healthy donors (Fig. 8a). However, the relation between the age of the donor and EGR1 expression identified in healthy donors was not confirmed in samples of AML patients suggesting a perturbation of this relation in this pathological context. Expression of EGR1 was confirmed to be dysregulated in AML LT-HSC and AML ST-HSC as compared to healthy donor samples comprising equivalent cell compartments from cord blood and bone marrow (Fig. 8c). Xeno-transplantation experiments showed that EGR1 higher expression was conserved in the bone marrow of the mouse especially with LT-HSC compartment as compared to ST-HSC and GMP compartments (Fig. 8c). In AML samples, overexpression of EGR1 did not concern pathological clones of leukemic stem cells (LSC) and leukemic progenitor cells (LPC) (Fig. 8c). Stratification AML cohort on karyotype status identified that high expression of EGR1 AML LT-HSC concerned patients with normal karyotype as compared to those which were affected by monosomy 7. These results suggest that EGR1 upregulation related to the age of donor in LT-HSC is effective in healthy donors this relation is perturbed in AML pathological context.

## Discussion

During aging, HSC harbor a decrease in regenerative properties associated to a myeloid-biased differentiation [37], a decrease of homing properties [38], and a platelet-biased differentiation [12]. Various stress factors could influence HSC aging like an increase of production of reactive oxygen species [37], an increase of DNA damage responses and replicative stress [39], as well as an increase of polarity through CDC42 regulation [40]. Also, the micro-environmental bone marrow niche is an important controller of HSC aging. A profound transcriptional perturbation associated with modification of chromatin access was recently confirmed at the single-cell level in aging HSC [21]. In the single-cell transcriptome of human HSC, we confirmed that older cells in this primitive compartment harbored perturbation of cell cycle components like CDK4/6/D-type cyclin CCND2 complex. These abnormal cells mainly lose expression of CDK6 which was found to be associated with a strong upregulation of the EGR1 transcription factor. This EGR1 upregulation in old HSC was found to be independent of the individual heterogeneity (Supplemental Figure 8). EGR1 is known to be upregulated in cyclin-dependent kinase 6 (CDK6) deficient HSC which do not efficiently repopulate in competitive transplantation assays and exhibit higher susceptibility to 5-fluorouracil (5FU) treatment. Activation of HSCs requires CDK6, a cell cycle regulator which interferes with some key transcription factors, including EGR1. EGR1 knockout in CDK6-/- BCR-ABL-p210 LSKs significantly enhances the potential to form colonies [35]. This activation of EGR1 in older HSC was found to be independent of the gender of the bone marrow donors. EGR1 is a member of the early growth response family and plays essential roles in cell growth, development, and stress responses in many tissues. EGR1 is also implicated in the myeloid lineage differentiation: c-fos and EGR1 represent key transcription factors that are differentially activated by macrophage colony-stimulating factor (M-CSF) and granulocyte colony-stimulating factor (G-CSF) to resolve neutrophil versus monocyte cell fate [41]. EGR1 contribute to the transcriptional control of monocytic differentiation. Egr-1, Egr-2, vitamin D receptor, MafB/c: Fos and PU.1:interferon regulatory factor 8 complexes direct further monocytic maturation, while retinoic acid receptor (RAR) and C/EBPepsilon direct granulopoiesis [42]. Gene expression analysis revealed that EGR1 and c-fos were downregulated in primitive hematopoietic primitive cells [43], but other studies suggested that EGR1 plays an indispensable role in regulating the homeostasis of HSCs [44].

EGR1^high^ old human HSCs harbor activation of quiescence markers: like EGR1, in older human HSC, we highlighted activation of BTG2, JUNB, and NR41A. In previously published LRP5/LRP6 double knockout model, there was an increase of quiescence in HSCs associated with an upregulation of quiesceace regulators such as EGR1, NR4A1, JUNB, and BTG2 [45]. BTG2 is an antiproliferative molecule [46] that we found to be overexpressed on aged HSCs which show disruption of CDK6/CCND2 cell cycle complex and high expression of EGR1. JUNB inactivation deregulates the cell cycle machinery and increases the LT-HSCs, and JunB protects against myeloid malignancies by limiting hematopoietic stem cell proliferation and differentiation without affecting self-renewal. Loss of the JunB/AP-1 transcription factor induces a myeloproliferative disease (MPD) known to arise  from HSC compartment. It was shown that junB inactivation deregulates the cell-cycle machinery and increases the proliferation of long-term repopulating HSCs (LT-HSCs) without impairing their self-renewal or regenerative potential in vivo [47]. Nr4a1 is preferentially expressed in HSCs and controls HSC quiescence by acting upstream of key HSC transcriptional networks. NR4A1 and NR4A3 restrict HSC proliferation via reciprocal regulation of C/EBPα and inflammatory signaling. Codepletion of NR4A1/3 promotes acute changes in HSC homeostasis including loss of HSC quiescence, accumulation of oxidative stress, and DNA damage while maintaining stem cell regenerative and differentiation capacity. NR4A1/3 restricts HSC proliferation in part through activation of a C/EBPα-driven anti-proliferative network by directly binding to a hematopoietic-specific Cebpa enhancer and activating Cebpa transcription. In addition, NR4A1/3 occupy the regulatory regions of NF-κB-regulated inflammatory cytokines, antagonizing the activation of NF-κB signaling [48].

In this study, we also found that some transmembrane molecules were expressed on old human HSC as compared to younger ones. These observations could help for a better characterization of molecular membrane patterns during aging. Among these molecules, we observed an increased expression of CD69, SELP, and TMEM107. In the old HSC human compartment, as compared to young ones, we highlighted an overexpression of CD69 that is known as an early activation marker that is expressed in hematopoietic stem cells and lymphoid cells [49]. During thymocyte development, it was shown that older mice have a reduced ability to respond to mitogens with a failure to upregulate expression of the activation marker CD69 and to enter the G(2)--M phase of the cell cycle [50]. Among alterations of the aged HSC phenotype, a myeloid-biased differentiation has been described [51]. In a recently described animal model of AML, leukemic stem cells characterized with a high expression of CD69 were found to be poorly proliferative and more capable self-renewal and [52], the same properties that we observed in the old human HSC compartment. TMEM107, a transmembrane molecule, is required for normal Sonic Hedgehog (shh) signaling in the development of the neural tube in combination with Gli2 and Gli3 to pattern ventral and intermediate neuronal cell types [53]. Sonic hedgehog signaling has been already shown to act on the proliferation of primitive human hematopoietic cells through bone morphogenic protein (BMP) regulation [54]. TMEM107 also belongs to the transition zone (TZ) ciliary subcompartment is thought to control cilium composition and signaling by facilitating a protein diffusion barrier at the ciliary base [55], so it could be an interesting candidate potentially implicated in cell polarity [56] and polarity dysfunction is implicated in dysregulated phenotype of aged HSCs [40].  Sonic hedgehog signaling plays an important role in the maintenance of symmetric cell division during neurogenesis [57]. In the human HSC co-expression module connected to overexpression of EGR1, we found SELP with a central connected role, as it is  shown to be specifically overexpressed in old LT-HSCs but it is not the case in less primitive compartments like ST-HSCs and MPPs [58].

In the old HSC human compartment, single-cell analysis confirmed an expression increased of inflammatory ligands such as IL1B and TNFSF10. In aged mice, interleukin 1B (IL1B) was found to be elevated in the bone marrow and caspase 1 activity, which can process pro-IL1B, was increased in marrow macrophages and neutrophils. This mechanism of IL1B signaling was necessary and sufficient to induce a platelet bias in HSCs [2]. TNFSF10 is implicated in TNF-related apoptosis-inducing ligand (TRAIL) at mitochondrial level [59] and interferes with response oxygen species (ROS) metabolism [60] which could be a stress for HSCs in relation to the level of oxygen in the bone marrow niche [61]. HIF1A hypoxic transcription factor expression was confirmed to higher in young HSC human compartment as compared to one from elderly donors. With similar connectivity to EGR1 in old HSC module, IER2 immediate early gene pip92 was confirmed to be upregulated in old HSC with one of the top predictive scores found by machine learning. IER2 is known to be induced downstream MAPK signaling in environmental stress condition [62]. IER proteins like IER2 are target protein-specific regulators of PP2A activity [63] from which its activity could eradicate tyrosine kinase inhibitor-resistant chronic myeloid leukemic stem cells [64].

In gene-connected module of the old HSC human compartment, we observed an increase of MYB oncogene and confirmed at single-cell level EGR1^HIGH^ HSC from elderly donors. MYB is known to suppress apoptosis in acute myeloid leukemia cells by transcriptional repression of DRAK2 alias STK17B [65]. High expression of STK17B was found with a central connect role in gene module of young HSC human compartment.

In contrast, young HSC human compartment was found with important expression of PDE4B a molecule implication in immune activation and potentially targetable by inhibitors such as Roflumilast used to correct inflammatory or autoimmune diseases [66]. Also, in young HSC human enrichment, we observed a higher expression of MYADM as compared to the ones of elderly donors. MYADM is a membrane protein with multiple putative transmembrane-spanning domains known to be expressed during myeloid differentiation [67]. This observation could be linked to the myeloid bias observed in aging hematopoiesis, but surprisingly, this myeloid marker was found repressed with age on HSCs.

## Conclusions

In conclusion, we report for the first time in this work that EGR1 is connected to the aging of human HSC. We describe a specific cell trajectory contributing to the dysregulation of an inflammatory and leukemia-related transcriptional program in aged human HSCs. This program was found with dissociation of quiescent and cell cycle phase progression in this primitive hematopoietic compartment. EGR1 upregulation was found in aged human hematopoietic stem cells independently of cell cycle phase progression, but these cells harbored cell cycle perturbations with principal loss of CDK6 and CCND2 during S phase. EGR1 high expression in aged human hematopoietic stem cells was also found to be associated with the activation of several quiescent markers like NR4A1, JUNB, and BTG2. EGR1 aged-related connected module harbored a gene network known to be implicated in the pathophysiology of leukemia and immune disorders.

## Supplementary Information

**Additional file 1: Supplemental figure 1.** Single cell expression analyses of markers belonging to the EGR1 cluster trajectory during aging of HSC. **Supplemental figure 2.** EGR1 cluster found on aging HSC trajectory is enriched in hematopoietic disorder signatures. **Supplemental figure 3.** EGR1 cluster found on aging HSC trajectory is enriched in immune disorder signatures. **Supplemental figure 4.** markers found to be up regulated in gene module of young HSC. **Supplemental figure 5.** single cell expression of markers found to be up regulated in gene module of young HSC. **Supplemental figure 6.** single cell expression of markers found to be up regulated in gene module of old HSC. **Supplemental figure 7.** cross batch normalization of human hematopoietic stem transcriptomes. **Supplemental figure 8.** Donor heterogeneity of EGR1 expression in human HSCs. **Supplemental table 1.** Best one hundred genes found to be significant on EGR1 cell trajectory inside human hematopoietic stem cells. **Supplemental table 2.** Best one hundred genes found to be differentially expressed by RNA-sequencing in dataset GSE104406 comparing young and old human hematopoietic stem cells. **Supplemental table 3.** Gene network connected in young HSCs turquoise module. **Supplemental table 4.** Gene network connected in old HSCs grey module.

## Data Availability

Datasets analyzed in this manuscript: - Single-cell transcriptome of human hematopoietic stem cell-enriched compartment (GSE104379): dataset link on NCBI website available at the address: https://www.ncbi.nlm.nih.gov/geo/query/acc.cgi?acc=GSE104379 (accessed on 19 January 2021) publish in the manuscript: Adelman ER, Huang HT, Roisman A, Olsson A et al. Aging human hematopoietic stem cells manifest profound epigenetic reprogramming of enhancers that may predispose to leukemia. Cancer Discov 2019 Aug;9(8):1080-1101. DOI: 10.1158/2159-8290.CD-18-1474 - Transcriptome RNA sequencing of human hematopoietic stem cell (GSE104406): dataset link on NCBI website available at the address: https://www.ncbi.nlm.nih.gov/geo/query/acc.cgi?acc=GSE104406 (accessed on 19 January 2021) publish in the manuscript : Adelman ER, Huang HT, Roisman A, Olsson A et al. Aging human hematopoietic stem cells manifest profound epigenetic reprogramming of enhancers that may predispose to leukemia. Cancer Discov 2019 Aug;9(8):1080-1101. DOI: 10.1158/2159-8290.CD-18-1474.

## Abbreviations

5FU: 5-Fluorouracil
ABCC4: ATP-binding cassette subfamily C member 4
ANGPT1: Angiopoietin 1
AP-1: Activator protein 1
ATP2B1: ATPase plasma membrane Ca2+ transporting 1
BCR-ABL: Translocation between BCR and ABL1 to form Philadelphia chromosome
BMP: Bone morphogenetic protein
BTG2: BTG anti-proliferation factor 2
CARD8: Caspase recruitment domain family member 8
CCND2: Cyclin D2
CD34: Cluster of differentiation 34
CD38: Cluster of differentiation 38
CD69: Cluster of differentiation 69
CDC42: Cell division cycle 42
CDK6: Cyclin-dependent kinase 6
CEBPA: CCAAT enhancer-binding protein alpha
DRAK2: DAP kinase-related apoptosis-inducing protein kinase 2
DEG: Differential expressed genes
DisGeNet: Database of gene-disease associations
DNA: Desoxyribonucleic acid
DUSP1: Dual specificity phosphatase 1
DYNLT1: Dynein light chain Tctex-type 1
EGR1: Early growth response 1
FOS: FBJ murine osteosarcoma viral oncogene homolog
FOSB: FBJ murine osteosarcoma viral oncogene homolog B
G-CSF: Granulocyte-colony stimulating factor
Gli2: Glioma-associated oncogene family zinc finger 2
GMP: Granulocyte myeloid progenitor
GSTM1: Glutathione S-transferase Mu 1
HG19: Human genome version 19
HIF1A: Hypoxia-inducible factor 1 subunit alpha
HNRNPH2: Heterogeneous nuclear ribonucleoprotein H2
HSC: Hematopoietic stem cell
HSC: Hematopoietic stem cell
IER: Immediate early response
IER2: Immediate early response 5
IL16: Interleukin 16
IL1B: Interleukin 1B
JUNB: Transcription factor Jun-B
KIAA0232: Uncharacterized protein KIAA0232
KLF4: Kruppel-like factor 4
LRP5: LDL receptor-related protein 5
LRP6: LDL receptor-related protein 6
LPC: Leukemic progenitor cell
LSC: Leukemic stem cell
LSK: Lineage-negative, SCA-positive, KIT-positive cells
LT-HSC: Long-term hematopoietic stem cell
MAPK: Mitogen-activated protein kinase
M-CSF: Macrophage colony-stimulating factor 1
MICA: MHC class I polypeptide-related sequence A
MIR1248: Micro-ribonucleic acid 1248
MPP: Multipotent progenitors
MYADM: Myeloid-associated differentiation marker
MYB: V-Myb avian myeloblastosis viral oncogene homolog
NFE2: Nuclear factor, erythroid 2
NR4A1: Nuclear receptor subfamily 4 group A member 1
NR4A3: Nuclear receptor subfamily 4 group A member 3
PDE4B: Phosphodiesterase 4B
PNP: Purine nucleoside phosphorylase
PP2A: Protein phosphatase 2 phosphatase activator
PTGS2: Prostaglandin-endoperoxide synthase 2
RAR: Retinoic acid receptor
RNAseq: Ribonucleic acid sequencing
ROS: Reactve oxygen species
SECISBP2: Selenocysteine insertion sequence-binding protein 2
SELP: Selectin P
shh: Sonic Hedgehog
ST-HSC: Short-term hematopoietic stem cell
STK17B: Serine/threonine kinase 17b, alias DRAK2
TMEM107: Transmembrane protein 107
TNFSF10: Tumor necrosis factor (ligand) superfamily, member 10
TP53INP1: Tumor protein P53 inducible nuclear protein 1
TPM: Transcript per million
TRAIL: TNF-related apoptosis-inducing ligand
TZ: Transition zone
VST: Variance stabilization transformation
WGCNA: Weighted correlation network analysis
ZC3H6: Zinc finger CCCH-type containing 6
ZFP36: Zinc finger protein 36, C3H type, homolog
