# Supplementary material for: EGR1 dysregulation defines an inflammatory and leukemic program in cell trajectory of human-aged hematopoietic stem cells (HSC)
Source: Stem Cell Res Ther. 2021 Jul 22;12:419. doi: 10.1186/s13287-021-02498-0 (PMC8296523; doi:10.1186/s13287-021-02498-0)
Supplement: Supplementary file 1 — Additional file 1: Supplemental figure 1. Single cell expression analyses of markers belonging to the EGR1 cluster trajectory during aging of HSC. Supplemental figure 2. EGR1 cluster found on aging HSC trajectory is enriched in hematopoietic disorder signatures. Supplemental figure 3. EGR1 cluster found on aging HSC trajectory is enriched in immune disorder signatures. Supplemental figure 4. markers found to be up regulated in gene module of young HSC. Supplemental figure 5. single cell expression of markers found to be up regulated in gene module of young HSC. Supplemental figure 6. single cell expression of markers found to be up regulated in gene module of old HSC. Supplemental figure 7. cross batch normalization of human hematopoietic stem transcriptomes. Supplemental figure 8. Donor heterogeneity of EGR1 expression in human HSCs. Supplemental table 1. Best one hundred genes found to be significant on EGR1 cell trajectory inside human hematopoietic stem cells. Supplemental table 2. Best one hundred genes found to be differentially expressed by RNA-sequencing in dataset GSE104406 comparing young and old human hematopoietic stem cells. Supplemental table 3. Gene network connected in young HSCs turquoise module. Supplemental table 4. Gene network connected in old HSCs grey module. [file 13287_2021_2498_MOESM1_ESM.docx]

**EGR1 dysregulation defines an inflammatory and leukemic program in cell trajectory of human aged hematopoietic stem cells (HSC)**

**SUPPLEMENTAL MATERIAL**

**Supplemental figure legends**

**Supplemental figure 1: Single cell expression analyses of markers belonging to the EGR1 cluster trajectory during aging of HSCe:** Single cell expression of some markers (CD69, JUNB, NFKBIA, DUSP1, BTG2, TMEM107, MIR1248, HSPA1B, NR4C1) found in EGR1 cell trajectory cluster in human HSCe and stratified on age of donors (Fisher exact test was employed between amount of positive cells in each groups, numbers of positive cells and mean expression of each marker are written in corresponding color of groups)

**Supplemental figure 2: EGR1 cluster found on aging HSCe trajectory is enriched in hematopoietic disorder signatures:** A/ Functional enrichment performed on DisGeNet database with genes found in EGR1 HSCe trajectory during ageing and enriched in hematopoietic disorders signatures, number of genes and negative logarithm 10 of False discovery rate q-values; B/ Functional enrichment network of EGR1 cluster genes found to be implicated in hematopoietic disorders with DisGeNet database.

**Supplemental figure 3: EGR1 cluster found on aging HSCe trajectory is enriched in immune disorder signatures:** A/ Functional enrichment performed on DisGeNet database with genes found in EGR1 HSCe trajectory during ageing and enriched in immune disorders signatures, number of genes and negative logarithm 10 of False discovery rate q-values; B/ Functional enrichment network of EGR1 cluster genes found to be implicated immune disorders with DisGeNet database.

**Supplemental figure 4: markers found to be up regulated in gene module of young HSCe:** A/Expression plot of best predictive markers found by machine learning in RNA-sequencing of HSCe (dataset GSE104406) ; B/ Single cell expression of some markers (KLF6, HIF1A) found in module of young HSCe and stratified on age of donors (Fisher exact test was employed between amount of positive cells in each groups, numbers of positive cells and mean expression of each marker are written in corresponding color of groups)

**Supplemental figure 5: single cell expression of markers found to be up regulated in gene module of young HSCe:** Single cell expression of some markers (TP53INP1, KIAA0232, HNRNPH2, SECISBP2, DYNLT1 and ATP2B1) found in module of young HSCe and stratified on age of donors (Fisher exact test was employed between amount of positive cells in each groups, numbers of positive cells and mean expression of each marker are written in corresponding color of groups)

**Supplemental figure 6: single cell expression of markers found to be up regulated in gene module of old HSCe:** Single cell expression of some markers (DUSP6, NFE2, ANGPT1, PTGS1, CARD8, ZC3H6) found in module of old HSCe and stratified on age of donors (Fisher exact test was employed between amount of positive cells in each groups, numbers of positive cells and mean expression of each marker are written in corresponding color of groups)

**Supplemental figure 7: cross batch normalization of human hematopoietic stem transcriptomes:** transcriptome experiments from distinct datasets were collected (GSE35008, GSE35010, GSE58299, GSE24006) and they are cross batch normalized with Combat algorithm taking in account disease and organs surrogates for bias correction.

**Supplemental figure 8: Donor heterogeneity of EGR1 expression in human HSCs**: A/ tSNE dimension reduction of human HSCs single cell transcriptome stratified by age groups: young versus old, B/ tSNE dimension reduction of human HSCs single cell transcriptome stratified by individual donors, C/ Ridgeplot of EGR1 expression stratified by individual donors, D/ Violinplot of EGR1 expression stratified by individual donors

**Supplemental figures**

**Supplemental figure 1**

**Supplemental figure 2**

**Supplemental figure 3**

**Supplemental figure 4**

**Supplemental figure 5**

**Supplemental figure 6**

**Supplemental figure 7**

**Supplemental Figure 8**

**Supplemental Tables**

**Supplemental table 1: Best one hundred genes found to be significant on EGR1 cell trajectory inside human hematopoietic stem cell:** table describes gene names p and q values of their significance on EGR1 cell trajectory, also number of cell positive for the expression of these markers.

**Supplemental table 2: Best one hundred genes found to be differentially expressed by RNA-sequencing in dataset GSE104406 comparing young and old human hematopoietic stem cells:** columns describe differential expression analysis with gene names, Log2 fold change, average expression, t Student statistics test, p-values, adjust p-values.

**Supplemental table 3: Gene network connected in young HSCs turquoise module:** columns describe firstly score obtained in PAMR machine learning algorithm and follow with some corresponding network parameters

**Supplemental Table 4: Gene network connected in old HSCs grey module:** columns describe firstly score obtained in PAMR machine learning algorithm and follow with some corresponding network parameters

**SUPPLEMENTAL MATERIAL**

**Supplemental Tables**

| **sc-RNAseq EGR1 cell trajectory** | **p-values** | **corrected q-values** | **number of cells with positive expression (n=650)** |
| --- | --- | --- | --- |
| **EGR1** | **0** | **0** | **440** |
| **HIST1H2AE** | **9.95579194619455E-117** | **6.64101101770907e-113** | **147** |
| **NR4A1** | **4.04260572518449E-106** | **1.79774676598954e-102** | **167** |
| **HIST1H2BG** | **6.7002562921831E-102** | **2.23470297985037e-98** | **243** |
| **IER2** | **4.3326174527451E-101** | **1.15602898874145e-97** | **199** |
| **HSPA1B** | **2.72675009404942E-85** | **6.06292883411889e-82** | **144** |
| **JUN** | **1.5655054113413E-84** | **2.98362967038633e-81** | **388** |
| **JUNB** | **2.64417727980382E-80** | **4.40949613623284e-77** | **186** |
| **HIST1H2BC** | **8.31781416665374E-67** | **1.23297731997031e-63** | **187** |
| **ZFP36** | **1.62244821269694E-64** | **2.16450816055899e-61** | **308** |
| **FOSB** | **2.07056474394717E-64** | **2.51121856809084e-61** | **463** |
| **HSPA1A** | **2.56973715905951E-63** | **2.85690528658441e-60** | **219** |
| **HIST2H2AA4** | **1.52774870139227E-59** | **1.5678227250211e-56** | **452** |
| **HIST2H2AA3** | **3.14159777463517E-59** | **2.99371827938627e-56** | **452** |
| **HIST1H3D** | **1.49494415000004E-48** | **1.32960332701004e-45** | **133** |
| **PTGS2** | **1.02868920645411E-47** | **8.57733918956519e-45** | **174** |
| **CD69** | **1.88785238103615E-47** | **1.48151991855313e-44** | **390** |
| **BTG2** | **4.77338431875725E-47** | **3.53787334425225e-44** | **267** |
| **DUSP1** | **1.73309939741148E-46** | **1.21690942425613e-43** | **546** |
| **HIST1H2AM** | **6.21926061649428E-46** | **4.14855779423251e-43** | **111** |
| **HIST1H4B** | **2.24513946634031E-41** | **1.42630502954505e-38** | **54** |
| **HIST1H3A** | **3.43354632630663E-40** | **2.08213370632985e-37** | **63** |
| **HIST1H3H** | **5.96604252431647E-37** | **3.46056405725679e-34** | **60** |
| **KLF4** | **6.55056899274922E-36** | **3.64129753884447e-33** | **138** |
| **MASTL** | **2.32180838544969E-35** | **1.23900982681137e-32** | **73** |
| **MYADM** | **2.93435489631538E-34** | **1.5056626412209e-31** | **407** |
| **IER5** | **4.99637371259605E-34** | **2.46876376665718e-31** | **64** |
| **HIST1H2BN** | **6.59311134250471E-34** | **3.14138208644126e-31** | **65** |
| **TMEM107** | **1.35826198176943E-33** | **6.24847348233998e-31** | **445** |
| **NFKBIA** | **1.76425839674208E-33** | **7.84565709031202e-31** | **353** |
| **DNAJB1** | **3.92413782087438E-33** | **1.68877169897694e-30** | **245** |
| **POLR3E** | **2.46933013324664E-32** | **1.02947916586386e-29** | **182** |
| **IGJ** | **3.04064904381244E-32** | **1.22925148162126e-29** | **67** |
| **MIR1248** | **5.41131933578853E-32** | **2.12330621349279e-29** | **302** |
| **PRSS3P2** | **9.08354294530504E-32** | **3.46238704095184e-29** | **39** |
| **PRSS1** | **5.0586234284425E-31** | **1.87464153219032e-28** | **37** |
| **CD83** | **1.72571623232185E-30** | **6.22237304200157e-28** | **84** |
| **HIST1H2BB** | **7.25323158608622E-30** | **2.54645691026253e-27** | **55** |
| **PRSS3** | **1.3568844284356E-29** | **4.64158850250239e-27** | **39** |
| **HSPH1** | **2.01860082651413E-29** | **6.73253840663125e-27** | **234** |
| **GSTM1** | **1.31248745814456E-27** | **4.2707061412455e-25** | **187** |
| **HIST1H2AD** | **7.50239476760817E-26** | **2.38308210939668e-23** | **70** |
| **HIST1H4E** | **7.97915263873294E-26** | **2.47557849658921e-23** | **101** |
| **PNP** | **4.62792574889109E-25** | **1.40320812308991e-22** | **108** |
| **CCR7** | **5.43204157136405E-25** | **1.61041925785706e-22** | **27** |
| **EGR3** | **7.19767697746976E-25** | **2.08748279470487e-22** | **41** |
| **SAT1** | **1.48304537513945E-24** | **4.2096400744118e-22** | **472** |
| **SNORA31** | **2.31111402244728E-24** | **6.4234525361394e-22** | **21** |
| **HIST1H4K** | **2.43504381170337E-24** | **6.62977948814992e-22** | **38** |
| **NPTX2** | **4.02731143970983E-24** | **1.07456723834338e-21** | **15** |
| **HIST1H4H** | **6.40802112287115E-24** | **1.67626293725929e-21** | **22** |
| **PDE4B** | **6.6350885725674E-24** | **1.70228301243503e-21** | **142** |
| **HIST1H2BD** | **6.99574845850276E-24** | **1.76094868273369e-21** | **281** |
| **ARRDC3** | **2.28131929395013E-23** | **5.63612605566456e-21** | **559** |
| **HIST1H2BL** | **1.2958750571748E-22** | **3.14332166141255e-20** | **34** |
| **SLC38A1** | **1.33400192512607E-22** | **3.17802137198337e-20** | **153** |
| **CCNH** | **1.37930426313492E-22** | **3.2282979253479e-20** | **255** |
| **FLT3LG** | **1.79912963272079E-22** | **4.13830835002208e-20** | **21** |
| **RPPH1** | **8.38705914769476E-22** | **1.89647044219315e-19** | **58** |
| **PHLDB2** | **1.09474013462539E-21** | **2.43415468933956e-19** | **205** |
| **PRKG1** | **1.33974416377661E-21** | **2.93008637523667e-19** | **87** |
| **FCER1A** | **2.1681989777196E-21** | **4.66547460673503e-19** | **306** |
| **HIST1H2AL** | **2.66613131825184E-21** | **5.64585046298379e-19** | **24** |
| **SLC2A3** | **9.02534837697723E-21** | **1.88136207339458e-18** | **169** |
| **ZFAND5** | **2.13585782269335E-20** | **4.3488862694942e-18** | **406** |
| **TAGAP** | **2.15146161297217E-20** | **4.3488862694942e-18** | **190** |
| **TMEM38B** | **2.77710098974428E-20** | **5.52974691107141e-18** | **166** |
| **SNORD47** | **3.20514369732694E-20** | **6.28820912735863e-18** | **45** |
| **HIST2H2AC** | **3.36453484494639E-20** | **6.505254980642e-18** | **71** |
| **HIST1H4F** | **3.66954352336313E-20** | **6.99362573502679e-18** | **22** |
| **MAFF** | **4.03042234246826E-20** | **7.57322034800972e-18** | **193** |
| **TRIB2** | **4.26948281221648E-20** | **7.91099586080278e-18** | **19** |
| **AGAP3** | **5.06497756521175E-20** | **9.19502650725589e-18** | **62** |
| **FOS** | **5.10030703498191E-20** | **9.19502650725589e-18** | **632** |
| **ATF3** | **5.61626005054406E-20** | **9.99020337790778e-18** | **69** |
| **HSPB1** | **6.27561797018598E-20** | **1.10161867552962e-17** | **404** |
| **CCNL1** | **1.38019434593276E-19** | **2.39132113884272e-17** | **492** |
| **GPM6B** | **1.4265251141748E-19** | **2.43990660874436e-17** | **25** |
| **DMKN** | **2.16624721649709E-19** | **3.6582157107959e-17** | **17** |
| **BTG1** | **3.61613085600632E-19** | **6.03035021874755e-17** | **241** |
| **TEPP** | **4.1790766005132E-19** | **6.88309394166008e-17** | **39** |
| **SOCS3** | **5.72961099265843E-19** | **9.32179759183611e-17** | **107** |
| **CNOT6** | **8.28065212779011E-19** | **1.33099012092588e-16** | **122** |
| **ZNF559** | **1.27885880537994E-18** | **2.03110182411593e-16** | **131** |
| **DUSP2** | **1.67304353454069E-18** | **2.62589103462439e-16** | **44** |
| **FBXO22** | **1.83560252248336E-18** | **2.84753177354076e-16** | **60** |
| **HSD17B10** | **3.2896546979946E-18** | **5.04451532482138e-16** | **143** |
| **LOC284454** | **4.41098822645155E-18** | **6.68715840103296e-16** | **633** |
| **ID2** | **4.87377447549897E-18** | **7.305733177262e-16** | **224** |
| **RNASET2** | **5.18578846415239E-18** | **7.68706710002856e-16** | **104** |
| **VTRNA1-1** | **5.38951194173501E-18** | **7.90126140820733e-16** | **55** |
| **SNORA19** | **7.25069086322259E-18** | **1.05142898702449e-15** | **26** |
| **CDCP1** | **1.48166011852002E-17** | **2.12546533776082e-15** | **322** |
| **IL8** | **1.85382504481477E-17** | **2.63105105562487e-15** | **42** |
| **ID1** | **7.34388310045034E-17** | **1.03131309940114e-14** | **51** |
| **HIST1H2BM** | **8.36371714080542E-17** | **1.1622953164113e-14** | **12** |
| **GSTT1** | **1.07653437640019E-16** | **1.4785874523507e-14** | **83** |
| **ATP6V1D** | **1.08613724855984E-16** | **1.4785874523507e-14** | **75** |
| **PIGU** | **1.11251227317218E-16** | **1.49919456933233e-14** | **62** |
| **COLQ** | **1.78530735182708E-16** | **2.38177853807251e-14** | **22** |

**Supplemental table 1: Best one hundred genes found to be significant on EGR1 cell trajectory inside human hematopoietic stem cell:** table describes gene names p and q values of their significance on EGR1 cell trajectory, also number of cell positive for the expression of these markers.

| **gene** | **logFC Old/Young HSCs** | **AveExpr** | **t** | **P.Value** | **adj.P.Val** |
| --- | --- | --- | --- | --- | --- |
| **PDE4B** | **-2.6135953688** | **10.6689594846** | **-10.746595891409** | **2.35563954632332E-11** | **3.30307777185456E-07** |
| **LMNA** | **-2.8586169817** | **11.07447151415** | **-10.3604815121014** | **5.31722156020095E-11** | **3.72790403585689E-07** |
| **MYADM** | **-1.36103501** | **11.491403896** | **-8.67174076296791** | **2.34070812936359E-09** | **0.0000109404697966454** |
| **FOSL2** | **-1.901921487** | **12.7979557255** | **-8.45339272950123** | **3.92395994406888E-09** | **0.0000137554415839335** |
| **RASGEF1B** | **-1.4291642327** | **10.85129827665** | **-7.83984411816771** | **1.73292297956933E-08** | **0.0000485980920390424** |
| **FIGN** | **2.1776545254** | **7.0927647908** | **7.56296035239146** | **3.44195729393474E-08** | **0.0000804385419592549** |
| **IER2** | **1.279750171** | **12.1211062265** | **7.40734598314803** | **5.08328783568466E-08** | **0.000101825517188529** |
| **EMP1** | **-1.2555296983** | **10.10223532585** | **-7.29777972060828** | **6.70122911245554E-08** | **0.000117455793268565** |
| **MOK** | **-1.0038986634** | **7.9011051674** | **-6.93925826095293** | **1.67195058274718E-07** | **0.00026048990079201** |
| **LDLR** | **-1.9482822002** | **10.7491310049** | **-6.80061486198871** | **2.39060058433523E-07** | **0.000321531811521554** |
| **HIST2H2BC** | **1.1141346997** | **8.64268826395** | **6.77988352834101** | **2.52235767132869E-07** | **0.000321531811521554** |
| **STEAP2** | **0.9090162874** | **7.1133523412** | **6.67781725634281** | **3.28711451990012E-07** | **0.000370454285597468** |
| **SHFM1** | **-1.1750681591** | **8.43232842825** | **-6.66094984774728** | **3.43453552472335E-07** | **0.000370454285597468** |
| **BOLA1** | **1.619217675** | **8.6908016582** | **6.6120581205056** | **3.90094233670427E-07** | **0.000390707238894766** |
| **VIM** | **-0.882492254999997** | **15.1214745785** | **-6.57868405439067** | **4.25580462033276E-07** | **0.000397832615908707** |
| **CD97** | **-0.860777931** | **11.0045927835** | **-6.34429965288038** | **7.86898495387501E-07** | **0.000689618168895221** |
| **EFNA1** | **0.880154229900001** | **7.68052970105** | **6.30432486706611** | **8.74336170634269E-07** | **0.000690266294372137** |
| **LTBP1** | **1.4039508369** | **8.78448074275** | **6.28660043361415** | **9.16196777351122E-07** | **0.000690266294372137** |
| **KLF6** | **-1.052136348** | **13.035843214** | **-6.27877466366959** | **9.35320182076066E-07** | **0.000690266294372137** |
| **TUB** | **1.0143205792** | **7.8657403811** | **6.25366289013202** | **9.99459775807062E-07** | **0.000700721248818331** |
| **EGR1** | **2.017919141** | **11.7801432135** | **6.17214398830873** | **1.24006623175578E-06** | **0.000828009938175216** |
| **STK17B** | **-1.209019643** | **12.6976070355** | **-5.98044464061585** | **2.06406616177771E-06** | **0.00131556071456577** |
| **HIST1H2AH** | **1.5544656316** | **11.0503200032** | **5.86101407583617** | **2.83941058009979E-06** | **0.00165721881562969** |
| **CCDC9** | **-0.862379737299999** | **9.86593808275** | **-5.85597110878381** | **2.87797308447466E-06** | **0.00165721881562969** |
| **MYCN** | **1.345986205** | **12.0905031325** | **5.83293879806656** | **3.06093414670746E-06** | **0.00165721881562969** |
| **CASC10** | **0.8320756146** | **8.358213863** | **5.83148572629221** | **3.07286330098216E-06** | **0.00165721881562969** |
| **JOSD1** | **-0.893765656000001** | **11.377530584** | **-5.80778503711416** | **3.27420115905283E-06** | **0.00170040180193477** |
| **ZNF230** | **0.8378922786** | **7.6688789541** | **5.68858208076415** | **4.50795265111652E-06** | **0.00225751828835557** |
| **BTBD3** | **-0.714819962100001** | **10.13590208295** | **-5.65728106085878** | **4.90355714700323E-06** | **0.0023709544246648** |
| **RARA** | **-1.1096666355** | **10.30654809225** | **-5.58813181220543** | **5.90617203877614E-06** | **0.00276054481092397** |
| **SLITRK5** | **1.1931141428** | **9.1944796178** | **5.57136550504113** | **6.17893842762419E-06** | **0.00279487337523053** |
| **BHLHE41** | **0.8151578085** | **7.26755340715** | **5.53232454783369** | **6.86431031459722E-06** | **0.0029817189551727** |
| **SCN8A** | **0.688531493300001** | **7.39957819745** | **5.52312208039339** | **7.03671693968187E-06** | **0.0029817189551727** |
| **PHTF1** | **-1.082342806** | **11.194701957** | **-5.49138304345482** | **0.0000076655051657** | **0.0029817189551727** |
| **HIST1H2AG** | **1.089683257** | **11.2736875545** | **5.49092528461836** | **7.67497620493855E-06** | **0.0029817189551727** |
| **GVINP1** | **0.8222812825** | **10.40773269375** | **5.48889160087487** | **7.71719579811728E-06** | **0.0029817189551727** |
| **ARL4C** | **-1.9619234331** | **8.05004984655** | **-5.48172286443117** | **7.86789340617528E-06** | **0.0029817189551727** |
| **LARP1P1** | **0.9321475958** | **6.6556117405** | **5.46490622734415** | **8.23313423458619E-06** | **0.00302266306442223** |
| **AC008440.5** | **-1.0085861633** | **7.60595465055** | **-5.44674191469337** | **8.64684602694445E-06** | **0.00302266306442223** |
| **AHNAK** | **-0.689241581999999** | **14.909775725** | **-5.44349852970959** | **8.72288962213889E-06** | **0.00302266306442223** |
| **TRPC6** | **0.5169024476** | **5.9059647612** | **5.43329619091091** | **0.0000089665069889513** | **0.00302266306442223** |
| **CDH6** | **0.814644093** | **5.6853264351** | **5.42679016653397** | **9.12542492945093E-06** | **0.00302266306442223** |
| **SIK1** | **-1.9163796536** | **11.1902874702** | **-5.4183613485744** | **9.33553116002388E-06** | **0.00302266306442223** |
| **SURF6** | **0.849005590899999** | **10.17625245255** | **5.4064517165827** | **9.64072789085101E-06** | **0.00302266306442223** |
| **TDRKH** | **0.787154203000002** | **8.811284086** | **5.40213032740819** | **9.75393281697573E-06** | **0.00302266306442223** |
| **TEKT2** | **-0.525176513200001** | **6.1798834046** | **-5.39602979419786** | **9.91602488685086E-06** | **0.00302266306442223** |
| **DNAJB6** | **-0.812444094** | **12.532292217** | **-5.38798204906728** | **0.0000101340067991199** | **0.00302338390079275** |
| **LRIF1** | **0.8307085563** | **9.19403083445** | **5.37836209244394** | **0.0000104009069459551** | **0.00303836494158714** |
| **KLF10** | **-1.25762804** | **12.575818319** | **-5.35308205609124** | **0.0000111364741479446** | **0.00318684980617305** |
| **GFI1B** | **1.2970413168** | **9.8189491446** | **5.34430208250085** | **0.0000114039990383376** | **0.0031981374903114** |
| **TRIM68** | **0.577042483099999** | **9.30438196825** | **5.32975468592044** | **0.0000118615530358646** | **0.00326122934644888** |
| **ZNF33B** | **0.615558254000001** | **10.999744765** | **5.31252697691362** | **0.0000124273372806134** | **0.00335107929516848** |
| **USP42** | **-0.7241917686** | **10.3162110297** | **-5.29728505103089** | **0.0000129504799461691** | **0.00340702538607845** |
| **SELP** | **0.970530347700002** | **10.44046724315** | **5.28538539505978** | **0.0000133742436187587** | **0.00340702538607845** |
| **DYNLT1** | **-0.951601554600001** | **9.1239857941** | **-5.28294687007929** | **0.0000134627882191984** | **0.00340702538607845** |
| **ZNF616** | **1.0935118801** | **8.64982002275** | **5.27901716392994** | **0.0000136067195564394** | **0.00340702538607845** |
| **SOCS3** | **-1.7651035781** | **11.00681104445** | **-5.25712822886767** | **0.0000144371871923954** | **0.00353116030837443** |
| **SNHG1** | **0.632854531999999** | **10.933017374** | **5.25283018242985** | **0.0000146061402000939** | **0.00353116030837443** |
| **ARAP3** | **0.635160685200001** | **7.1241866048** | **5.24563779988202** | **0.0000148933207070799** | **0.00353956174499449** |
| **ZNF2** | **0.832790256799999** | **7.2423866135** | **5.2243756187466** | **0.0000157758731383185** | **0.0036612349730982** |
| **HIC2** | **-0.7110209295** | **9.77028665935** | **-5.22084337000878** | **0.0000159274948908137** | **0.0036612349730982** |
| **KBTBD3** | **0.5849208911** | **6.57979499355** | **5.2085558037683** | **0.0000164664257827288** | **0.00372406810202296** |
| **CD48** | **-0.943489697799999** | **8.336121739** | **-5.18811247063852** | **0.0000174039346354764** | **0.00386606152955147** |
| **ITGA2B** | **0.929375592799999** | **10.6799569586** | **5.18301985559919** | **0.0000176456951855152** | **0.00386606152955147** |
| **ANXA1** | **-0.811630494999999** | **12.6808897925** | **-5.16144765486136** | **0.0000187077254877162** | **0.00402954759888141** |
| **HIST1H2BB** | **1.9867642381** | **9.96730943495** | **5.14700080076114** | **0.0000194545888090528** | **0.00402954759888141** |
| **ANPEP** | **-0.8210551001** | **10.35578453295** | **-5.1379964377347** | **0.0000199351457080122** | **0.00402954759888141** |
| **NFIL3** | **-1.4505128844** | **10.2426845046** | **-5.13538662794403** | **0.0000200766420699207** | **0.00402954759888141** |
| **DDA1** | **-0.8220727778** | **9.040728719** | **-5.13365745489783** | **0.0000201709471582019** | **0.00402954759888141** |
| **HSPA2** | **0.489790665200001** | **6.1579046587** | **5.12793113434337** | **0.0000204864304007495** | **0.00402954759888141** |
| **SYAP1** | **-1.0946725455** | **10.29214013625** | **-5.12534562742784** | **0.0000206304919733822** | **0.00402954759888141** |
| **BHLHE40** | **-1.4761130876** | **10.8449898322** | **-5.12274813175224** | **0.000020776244480306** | **0.00402954759888141** |
| **ARHGAP6** | **0.6741003302** | **8.179689672** | **5.11683653730908** | **0.0000211118206763198** | **0.00402954759888141** |
| **BCL3** | **-1.7688309241** | **9.04459155725** | **-5.10857355408795** | **0.0000215900019715546** | **0.00402954759888141** |
| **HIST1H2AB** | **1.3793796104** | **9.4827432911** | **5.10674125569378** | **0.0000216975000094759** | **0.00402954759888141** |
| **CDKN2AIP** | **0.8452100832** | **10.4003076164** | **5.10432014049648** | **0.0000218403663896012** | **0.00402954759888141** |
| **SEC61B** | **-0.7292232628** | **8.3905194962** | **-5.09581219265158** | **0.0000223499287340509** | **0.00407000910011508** |
| **PER1** | **-1.231955296** | **13.297628937** | **-5.03483873946689** | **0.0000263677785715741** | **0.00474011527090528** |
| **PDE1C** | **1.087021269** | **8.7010445292** | **5.00123141592526** | **0.0000288838876888854** | **0.00507053785733528** |
| **TP53INP1** | **-0.961015348199999** | **10.2553858769** | **-4.99922736980318** | **0.0000290413051115978** | **0.00507053785733528** |
| **NUDT16P1** | **0.5740431578** | **6.3847858181** | **4.99477896262496** | **0.0000293938027480253** | **0.00507053785733528** |
| **TMEM200A** | **0.801681926600001** | **8.9865764995** | **4.99155101820029** | **0.0000296522681715513** | **0.00507053785733528** |
| **PIM3** | **-1.1209537847** | **10.32483227165** | **-4.97968230664787** | **0.0000306223263689283** | **0.00510024131896861** |
| **SLA** | **1.0894159454** | **11.0937649713** | **4.97912943907477** | **0.0000306682799630881** | **0.00510024131896861** |
| **PTS** | **-0.7498844455** | **8.45721019545** | **-4.9734819848409** | **0.0000311416604457046** | **0.00510024131896861** |
| **RHOH** | **-0.59567468** | **10.599816357** | **-4.97183721768223** | **0.0000312808981194765** | **0.00510024131896861** |
| **EZR** | **-0.948254662000001** | **11.389720275** | **-4.96369819365328** | **0.0000319791307429706** | **0.00515187244134863** |
| **PRKG1** | **1.588695877** | **9.4772946373** | **4.95964788593453** | **0.0000323323901610811** | **0.00515187244134863** |
| **LRP1** | **0.700445118** | **10.938701356** | **4.94330929042922** | **0.0000337974995946193** | **0.00531418135023836** |
| **B3GNT5** | **-0.882091243** | **10.1474618022** | **-4.93989139239596** | **0.0000341122909927851** | **0.00531418135023836** |
| **VPS37B** | **-0.560487203999999** | **12.149345793** | **-4.92618491996299** | **0.0000354044111346877** | **0.00531418135023836** |
| **RAB11FIP1** | **-0.663050784999999** | **10.5104333445** | **-4.92477340554132** | **0.0000355402252315095** | **0.00531418135023836** |
| **NR4A3** | **-1.5822945477** | **9.29288938145** | **-4.92320736964592** | **0.000035691517059126** | **0.00531418135023836** |
| **KLF3** | **-0.787184145** | **11.4576614415** | **-4.91702977277645** | **0.0000362946317491287** | **0.00531418135023836** |
| **SRGN** | **-0.807862008999999** | **11.5984672405** | **-4.91582426735759** | **0.0000364135076688888** | **0.00531418135023836** |
| **ECHDC1** | **0.683427438400001** | **8.9435846893** | **4.91467356503355** | **0.0000365273426263028** | **0.00531418135023836** |
| **MICA** | **0.609050469900001** | **8.92420304275** | **4.91231364008746** | **0.0000367619163438255** | **0.00531418135023836** |
| **UBE2J1** | **-0.552541923000001** | **11.3463266655** | **-4.89779538466168** | **0.0000382385208803556** | **0.00547122999779945** |
| **SCD** | **-0.746046123699999** | **8.72978068885** | **-4.887240069922** | **0.0000393491632839777** | **0.00557327239967612** |
| **DCTN2** | **-0.576199215999998** | **11.025576922** | **-4.87519933092084** | **0.0000406555320127375** | **0.00570000678291156** |

**Supplemental table 2: Best one hundred genes found to be differentially expressed by RNA-sequencing in dataset GSE104406 comparing young and old human hematopoieitic stem cells:** columns describe differential expression analysis with gene names, Log2 fold change, average expression, t Student statistics test, p-values, adjust p-values.

| **gene (turquoise YOUNG HSCs)** | **ranking PAMR** | **PAMR Old_HSCs-score** | **PAMR Young_HSCs-score** | **NeighborhoodConnectivity** | **NumberOfDirectedEdges** |
| --- | --- | --- | --- | --- | --- |
| **PDE4B** | **1** | **-1.2185** | **1.2185** | **10.0** | **4** |
| **MYADM** | **2** | **-0.8395** | **0.8395** | **10.0** | **4** |
| **SOCS3** | **3** | **-0.6591** | **0.6591** | **25.5** | **2** |
| **STK17B** | **4** | **-0.6359** | **0.6359** | **18.5** | **12** |
| **KLF6** | **5** | **-0.623** | **0.623** | **4.0** | **2** |
| **VIM** | **6** | **-0.6004** | **0.6004** | **19.72727273** | **22** |
| **SYAP1** | **7** | **-0.5557** | **0.5557** | **21.40909091** | **22** |
| **DYNLT1** | **8** | **-0.5385** | **0.5385** | **17.0** | **2** |
| **TP53INP1** | **9** | **-0.5219** | **0.5219** | **22.0** | **15** |
| **RELL1** | **10** | **-0.504** | **0.504** | **25.44444444** | **9** |
| **SKIL** | **11** | **-0.4793** | **0.4793** | **20.95833333** | **24** |
| **NDUFV2** | **12** | **-0.4769** | **0.4769** | **20.56** | **25** |
| **SEC61B** | **13** | **-0.4765** | **0.4765** | **21.2** | **20** |
| **SLC2A3** | **14** | **-0.4657** | **0.4657** | **23.26315789** | **19** |
| **ATP2B1** | **15** | **-0.4521** | **0.4521** | **20.44** | **25** |
| **RAB11FIP1** | **16** | **-0.4501** | **0.4501** | **28.0** | **4** |
| **HIPK1** | **17** | **-0.4413** | **0.4413** | **17.61290323** | **31** |
| **TES** | **18** | **-0.4378** | **0.4378** | **23.0** | **2** |
| **ADNP2** | **19** | **-0.4126** | **0.4126** | **19.48148148** | **27** |
| **FAM91A1** | **20** | **-0.4123** | **0.4123** | **21.09090909** | **22** |
| **PPP1R2** | **21** | **-0.4108** | **0.4108** | **21.95** | **20** |
| **KIAA0232** | **22** | **-0.4092** | **0.4092** | **23.17647059** | **17** |
| **RNF125** | **23** | **-0.4086** | **0.4086** | **24.35714286** | **14** |
| **PPP1CB** | **24** | **-0.4024** | **0.4024** | **21.08333333** | **24** |
| **SUB1** | **25** | **-0.4017** | **0.4017** | **19.88461538** | **26** |
| **SIAH1** | **26** | **-0.3999** | **0.3999** | **26.1** | **10** |
| **MXD1** | **27** | **-0.3983** | **0.3983** | **23.47058824** | **17** |
| **F11R** | **28** | **-0.3866** | **0.3866** | **22.0** | **8** |
| **HIF1A** | **29** | **-0.3833** | **0.3833** | **20.19230769** | **26** |
| **U2AF1** | **30** | **-0.3797** | **0.3797** | **19.17857143** | **28** |
| **BLOC1S2** | **31** | **-0.3777** | **0.3777** | **5.5** | **2** |
| **ZC3H15** | **32** | **-0.3749** | **0.3749** | **23.8125** | **16** |
| **RNF10** | **33** | **-0.3745** | **0.3745** | **20.875** | **24** |
| **HNRNPH2** | **34** | **-0.3606** | **0.3606** | **22.8** | **10** |
| **UQCRFS1** | **35** | **-0.3558** | **0.3558** | **2.0** | **1** |
| **N4BP2** | **36** | **-0.3533** | **0.3533** | **20.6** | **25** |
| **SECISBP2** | **37** | **-0.3512** | **0.3512** | **22.57142857** | **21** |
| **RNF19A** | **38** | **-0.3442** | **0.3442** | **24.2** | **10** |

**Supplemental table 3: Gene network connected in young HSCs turquoise module:** columns describe firstly score obtained in PAMR machine learning algorithm and follow with some corresponding network parameters

| **gene (GREY OLD HSCs)** | **ranking PAMR** | **PAMR Old_HSCs-score** | **PAMR Young_HSCs-score** | **NeighborhoodConnectivity** | **NumberOfDirectedEdges** |
| --- | --- | --- | --- | --- | --- |
| **EGR1** | **1** | **0.7664** | **-0.7664** | **19.66666667** | **9** |
| **IER2** | **2** | **0.7413** | **-0.7413** | **18.625** | **8** |
| **CDH7** | **4** | **0.5444** | **-0.5444** | **28.42857143** | **28** |
| **SELP** | **5** | **0.5411** | **-0.5411** | **24.51724138** | **29** |
| **RPA2** | **6** | **0.5131** | **-0.5131** | **27.36666667** | **30** |
| **PCYOX1** | **9** | **0.4796** | **-0.4796** | **27.53333333** | **30** |
| **DUSP6** | **10** | **0.4758** | **-0.4758** | **27.43333333** | **30** |
| **JUN** | **11** | **0.4697** | **-0.4697** | **16.8** | **5** |
| **NFE2** | **12** | **0.4664** | **-0.4664** | **27.43333333** | **30** |
| **DDX60** | **13** | **0.4645** | **-0.4645** | **27.36666667** | **30** |
| **SNHG1** | **14** | **0.4595** | **-0.4595** | **25.07142857** | **28** |
| **ZNF33B** | **15** | **0.4585** | **-0.4585** | **26.5483871** | **31** |
| **IL16** | **16** | **0.4543** | **-0.4543** | **27.53333333** | **30** |
| **ZNF253** | **17** | **0.4484** | **-0.4484** | **26.62068966** | **29** |
| **PTGS1** | **18** | **0.4401** | **-0.4401** | **28.06896552** | **29** |
| **MICA** | **19** | **0.4342** | **-0.4342** | **19.5** | **10** |
| **PTAFR** | **21** | **0.4287** | **-0.4287** | **28.06896552** | **29** |
| **TCTEX1D1** | **22** | **0.4254** | **-0.4254** | **27.47368421** | **19** |
| **TNFSF10** | **24** | **0.4211** | **-0.4211** | **28.88** | **25** |
| **PRKG2** | **26** | **0.4181** | **-0.4181** | **27.86206897** | **29** |
| **ZC3H6** | **27** | **0.4177** | **-0.4177** | **28.48148148** | **27** |
| **MYB** | **28** | **0.4141** | **-0.4141** | **27.72413793** | **29** |
| **ANGPT1** | **30** | **0.4088** | **-0.4088** | **28.21428571** | **28** |
| **ABHD10** | **31** | **0.408** | **-0.408** | **27.20689655** | **29** |
| **IL1B** | **32** | **0.4059** | **-0.4059** | **28.44444444** | **27** |
| **ZNF470** | **34** | **0.3966** | **-0.3966** | **27.5** | **28** |
| **THAP5** | **37** | **0.3824** | **-0.3824** | **27.85714286** | **28** |
| **LARP4B** | **38** | **0.3699** | **-0.3699** | **27.36666667** | **30** |
| **UBE3B** | **40** | **0.3553** | **-0.3553** | **28.06896552** | **29** |
| **ABCC4** | **41** | **0.3499** | **-0.3499** | **28.88461538** | **26** |
| **TESPA1** | **42** | **0.347** | **-0.347** | **28.06896552** | **29** |
| **TMEM87B** | **43** | **0.3468** | **-0.3468** | **29.15789474** | **19** |
| **CARD8** | **44** | **0.3399** | **-0.3399** | **28.06896552** | **29** |

**Supplemental Table 4: Gene network connected in old HSCs grey module:** columns describe firstly score obtained in PAMR machine learning algorithm and follow with some corresponding network parameters
